# Generation and Characterization of Novel Low-Amylose Rice Germplasm via Prime Editing of the *Wx* Promoter

**DOI:** 10.3390/ijms27188386

**Published:** 2026-09-20

**Authors:** Jianju Liu, Yue Cai, Zichun Chen, Yunyu Wu, Peng Gao, Ling Yu, Wei Shi, Zhiping Wang, Shuhao Zhu, Cunhong Pan, Niansheng Huang, Yuhong Li, Xiaoxiang Zhang, Hongjuan Ji, Jianhua Zhao, Ning Xiao, Aihong Li

**Affiliations:** 1 Institute of Agricultural Sciences for Lixiahe Region in Jiangsu, Yangzhou Rice Experiment Station of the China Agricultural Research System, Yangzhou 225009, China; jianjuliu23@163.com (J.L.); cy890116@yeah.net (Y.C.); chenzi0109@outlook.com (Z.C.); wuyunyuyu@163.com (Y.W.); 18762329339@163.com (P.G.); jsyzyl_2007@126.com (L.Y.); shi-wei_nj2021@126.com (W.S.); lxhwzp0106@163.com (Z.W.); 15903637967@163.com (S.Z.); pancun-hong@163.com (C.P.); jsyzhns@163.com (N.H.); yhlirice@163.com (Y.L.); zhngyz@126.com (X.Z.); hongjuan-ji@163.com (H.J.); 20250076@jaas.ac.cn (J.Z.); 2 Collaborative Innovation Center for Modern Crop Production Co-Sponsored by Province and Ministry, Nanjing 210095, China; 3 Zhongshan Biological Breeding Laboratory, Nanjing 210014, China

**Keywords:** rice, amylose content, grain quality, promoter editing, *Wx*

## Abstract

Amylose content (AC) is primarily regulated by the *Wx* gene and acts as a key determinant of rice eating and cooking quality. Natural low-AC alleles are limited in fine-tuning effects across genetic backgrounds, and studies exploiting natural allelic variation are nearing saturation. Here, we employed prime editing (PE) to generate precise promoter deletions near the transcription start site (TSS) of *Wx* in *japonica* rice Huaidao 5, obtaining two homozygous mutant lines: *Wx^5^^d^* harboring the designed 5 bp deletion and *Wx^32d^* carrying an unintended 32 bp promoter deletion. Compared with the wild-type, AC was significantly reduced by 3.95 and 2.77 percentage points, respectively, with no obvious changes in grain transparency or the eight major agronomic traits evaluated in this study. The edited lines showed higher taste values measured by a Satake cooked rice taste analyzer, with *Wx^32d^* achieving the highest score (76.6 versus 72.6 for the wild-type). Gel consistency was correspondingly longer in both edited lines, and Rapid Visco Analyser (RVA) pasting profiles showed higher breakdown viscosity and more negative setback, most prominently in *Wx^32d^*. Reduced AC was accompanied by decreased *Wx* transcript accumulation and lower granule-bound starch synthase I (OsGBSSI) protein abundance. These two alleles were absent in 3032 natural rice accessions. This study illustrates that PE-mediated promoter editing can efficiently generate valuable low-amylose rice germplasm with minimal agronomic trade-offs among the traits evaluated under the tested environment, providing valuable intermediate materials for rice quality breeding.

## 1. Introduction

Rice (*Oryza sativa* L.) is a staple food crop worldwide, and improving grain quality remains a central objective in rice breeding programs [1,2,3]. Amylose content (AC) is the primary determinant of rice eating and cooking quality [4,5], largely controlled by the *Wx* gene, which encodes granule-bound starch synthase I (OsGBSSI). The expression level of *Wx* is tightly correlated with grain AC, and even subtle expression changes can significantly affect palatability and cooking properties [6].

To date, multiple low-AC alleles (AC ranging from 8% to 12%) caused by coding-sequence variations have been characterized, including *Wx^mp^* (~10%), *Wx^op/hp^* (~10–15%), *Wx^mq^* (~6–14%) and *Wx^mw^/Wx^la^* (~13%) [7,8,9,10,11]. Although these alleles confer superior palatability and have served as invaluable genetic resources [4,10,11,12,13,14,15,16,17], their practical application is constrained by a tendency to trigger excessive amylose reduction, alongside nearly exhausted allelic diversity, which limits opportunities for precise trait modulation [18]. While *Wx* promoter-targeted research has opened new avenues, naturally occurring promoter variants reported to date rarely provide elite alleles supporting stable and fine-tuned AC regulation, leaving a critical gap in strategies for improving rice eating and cooking quality. Notably, CRISPR/Cas9-mediated promoter editing of *Wx* has previously been shown to fine-tune AC and improve grain quality [18]; however, because NHEJ-based repair generates stochastic indels, large-scale screening is required to recover desired alleles, and precisely predefined promoter deletions remain difficult to obtain. A potential target for addressing this gap lies in the *Wx* promoter itself.

In plants, core promoter regions frequently contain pyrimidine-rich regions (PRRs) composed of CT/TC dinucleotide repeats, which are often located near the transcription start site (TSS) and may influence transcription levels [19,20,21,22]. The *Wx* promoter has been characterized in silico as a putative TATA-less promoter containing a PRR near its TSS [23]. Given that natural variation in this specific region appears limited among available rice germplasm collections, it represents an untapped target for generating novel alleles not accessible through conventional germplasm screening. Compared with the well-characterized intron-1 splicing strategy, which typically causes strong, stepwise reductions in AC, partial deletions within this core-promoter PRR were expected to modulate *Wx* transcription in a graded manner [24,25], thereby enabling fine-tuned AC reduction while preserving the elite genetic background of Huaidao 5.

The prime editing (PE) system, which enables precise small deletions without introducing double-strand breaks (DSBs) [26], provides an ideal tool for modifying such regulatory sequences. In this study, we applied PE to introduce defined deletions in the −11 to −7 bp region of the *Wx* promoter. This effort yielded, in addition to the intended 5 bp deletion, an unintended 32 bp deletion spanning two segments within the −45 to −7 region in the elite *japonica* variety Huaidao 5. We recovered two novel low-AC alleles, *Wx^32d^* and *Wx^5d^*, which reduced AC by 3.95 and 2.77 percentage points, respectively, without significant alterations in grain transparency or major agronomic traits under field conditions. Neither allele was detected among the 3032 surveyed rice accessions, suggesting these mutant alleles are absent from this panel of germplasm. This study delivers novel germplasm resources for breeding rice with reduced AC and raises the possibility that the −45 to −7 region of the *Wx* promoter might contain functionally relevant sequence elements worthy of further study.

## 2. Results

### 2.1. Screening of Homozygous Gene Editing Lines

PE-mediated transformation of Huaidao 5 yielded 15 independent T_0_ plants from 200 calli, corresponding to a transformation efficiency of 7.50%. Ten plants harbored target-site mutations, corresponding to a mutation efficiency of 66.67%. Two discontinuous deletion genotypes were obtained: four lines contained an unintended 32 bp deletion (designated *Wx^32d^*), and three lines harbored the intended precise 5 bp deletion (*Wx^5d^*). The remaining three lines exhibited complex heterogeneous indels and were excluded from subsequent analyses (Table 1). Homozygous T_2_ progenies for each deletion genotype were isolated.

**Figure A1:**
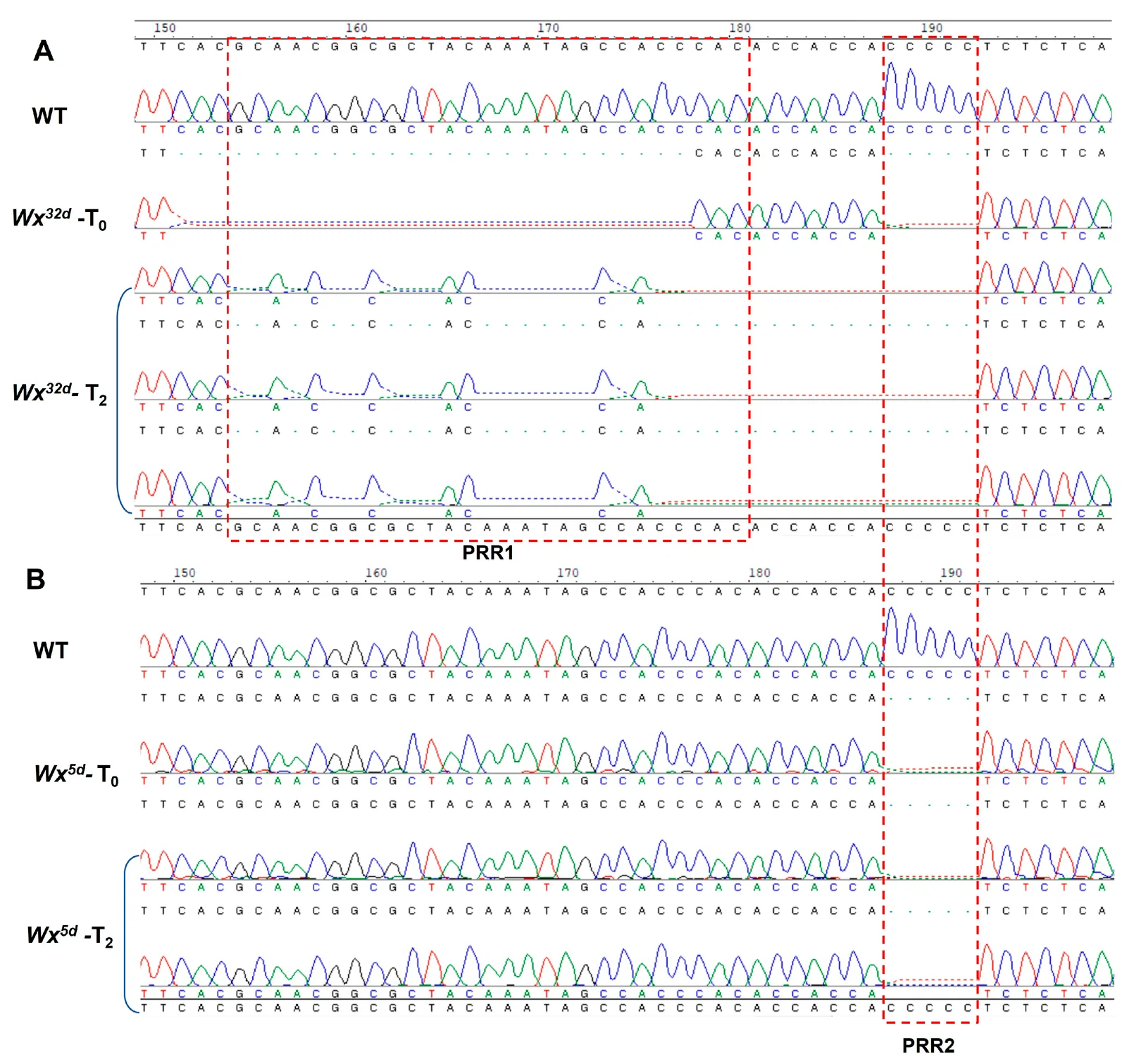
Representative Sanger sequencing chromatograms of the *Wx^32^^d^* and *Wx^5^^d^* edited lines in the T_0_ and T_2_ generations. Nucleotide peaks are color-coded as follows: green, adenine (A); red, thymine (T); blue, cytosine (C); black, guanine (G). Dashes in the aligned sequences indicate deleted nucleotides. Red dashed boxes mark the PRR1 and PRR2 regions.

The two alleles differ substantially in the extent of genomic deletion. The *Wx^32d^* allele carries a 32 bp deletion covering two discrete segments: −45 to −19 (27 bp) and −11 to −7 (5 bp), hereafter referred to as PRR1 and PRR2 for descriptive convenience (Figure A1). By contrast, *Wx^5d^* retains intact PRR1 and solely contains the 5 bp deletion within PRR2. This structural gradient enables us to examine whether deletion length correlates with the magnitude of phenotypic changes.

Editing specificity was assessed via Cas-OFFinder prediction combined with Sanger sequencing validation. Seven candidate off-target loci with ≤ three mismatches and DNA bulges were identified across the rice genome; no predicted sites contained zero or one mismatch. All candidate loci were located within intergenic regions or introns (Table A1). Sanger sequencing of the five highest-risk candidate sites in both edited lines revealed no detectable off-target mutations, supporting high editing fidelity of the PE system (Table A2). Representative Sanger sequencing chromatograms of the target amplicons from homozygous *Wx^5d^* and *Wx^32d^* plants are provided in Figure A1; clean single-peak traces across the deletion junctions, reproducible across independent plants and generations, support that the observed deletions are genuine editing products rather than PCR-induced artifacts.

### 2.2. Wx Novel Alleles Created by Prime Editing Are Extremely Rare in Natural Rice Populations

**Figure 1:**
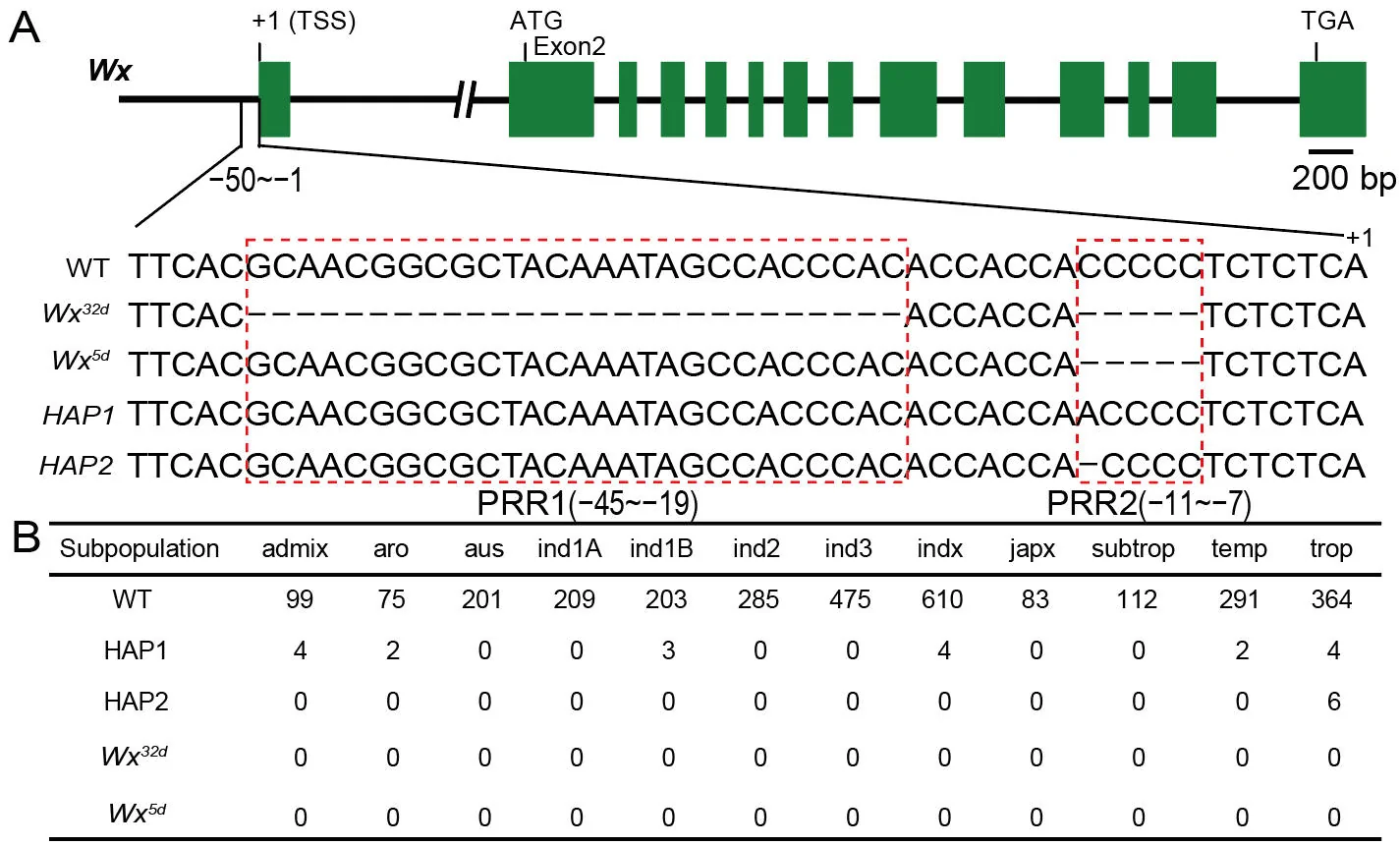
Two mutants obtained by precisely editing the core region of the *Wx* promoter using the PE system. (**A**) structure of the *Wx* gene and editing types of mutants; (**B**) variation types of natural germplasm in this region. The two red dashed rectangles delineate the sequences of PRR1 (−45 to −19 bp) and PRR2 (−11 to −7 bp), respectively.

To evaluate the prevalence of these edited sequence variants in natural populations, the promoter sequences of 3032 rice accessions from the SNP-Seek database (https://snp-seek.irri.org/, accessed on 15 September 2026) were analyzed. The target editing region was found to be highly conserved in natural populations: only 0.63% (19/3032) carried the HAP1 variant (C-to-A substitution at −11), and 0.20% (6/3032) carried the HAP2 variant (single C deletion at −11). The remaining accessions exhibited *Wx* promoter sequences identical to those of Huaidao 5 (Figure 1), indicating that neither of the two structural variations was detected among the 3032 accessions surveyed, suggesting that these alleles are rare or absent in this natural population panel.

### 2.3. Sequence Alterations Within the −45 to −7 Region of the Wx Promoter Are Associated with Reduced AC and Improved Eating Quality Parameter

**Figure 2:**
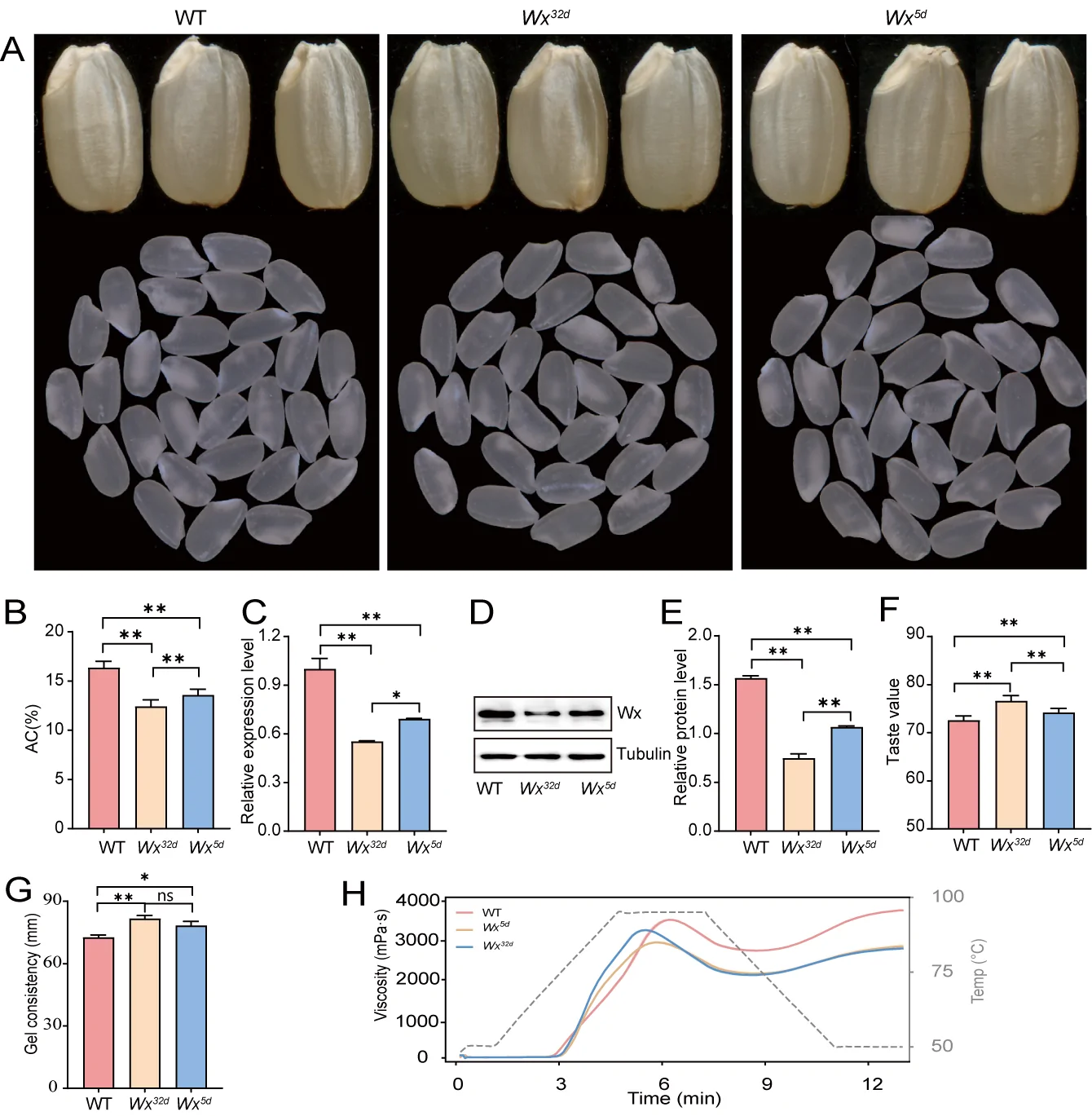
Grain appearance, transcriptional and translational levels of the *Wx*, eating-quality parameters, GC, and RVA pasting properties of the edited lines. (**A**) Comparison of brown and polished rice grain appearance between mutants and WT; (**B**) amylose content of mutants and WT; (**C**) relative expression level of *Wx* in mutants and WT; (**D**) Western blot results of mutants and WT; (**E**) relative protein expression in mutants and WT; (**F**) comparison of taste values between mutants and WT; (**G**) gel consistency of mutants and WT; (**H**) RVA pasting profiles of mutants and WT. Values in (**B**,**C**,**E**–**H**) represent mean ± SD (*n* = 3 biological replicates, each with three technical replicates). Statistical significance was determined by one-way ANOVA followed by Tukey’s HSD post hoc test. **, *p* < 0.01; *, *p* < 0.05; ns, not significant (*p* > 0.05). In (**D**,**E**), GBSSI protein levels were normalized against tubulin as the loading control.

AC quantification demonstrated that AC was significantly decreased in both mutant lines relative to the wild-type control. The AC of *Wx^32d^* decreased from 16.35% in the wild-type Huaidao 5 to 12.40% (a reduction of 3.95 percentage points), while *Wx^5d^* decreased to 13.58% (a reduction of 2.77 percentage points). Grain transparency showed no significant changes (Figure 2A,B). Quantitative measurements of grain chalkiness and translucency further supported the absence of significant differences between the edited lines and the wild-type (Table A3). Taken together, these results suggest that deletions within this region could contribute to reduced AC, which warrants further functional investigation of these sequences.

Molecular analyses showed that reduced AC was associated with decreased *Wx* transcription and OsGBSSI protein levels (Figure 2C–E). Taste value determination showed that the wild-type, *Wx*^32^*^d^*, and *Wx^5d^* had taste values of 72.6, 76.6, and 74.2, respectively. The *Wx^32d^* mutant with the lowest AC exhibited the highest taste value (Figure 2F), which is consistent with the general trend that moderate AC reduction improves rice eating quality in *japonica* rice. It should be noted that additional sensory evaluation is still required to validate the real edible quality of these materials. The taste values reported here should therefore be regarded as instrumental approximations of palatability.

Gel consistency (GC) was significantly longer in *Wx^32d^* (81.7 ± 1.5 mm) and *Wx^5d^* (78.3 ± 2.1 mm) than in WT (72.7 ± 1.2 mm; Figure 2G), with no significant difference between the two edited lines. RVA pasting profiles showed a consistent pattern (Table 2; Figure 2H): *Wx^32d^* had the highest breakdown viscosity (1007.3 ± 19.2 mPa·s) and the most negative setback (−468.0 ± 13.4 mPa·s), whereas WT had the lowest breakdown viscosity (601.8 ± 43.2 mPa·s) and a positive setback (+240.2 ± 19.9 mPa·s). These GC and RVA profiles are consistent with the reduced AC and higher instrumental taste values of the edited lines.

### 2.4. Prime Editing Targeting the Wx Promoter Does Not Cause Significant Changes to Major Agronomic Traits

**Figure 3:**
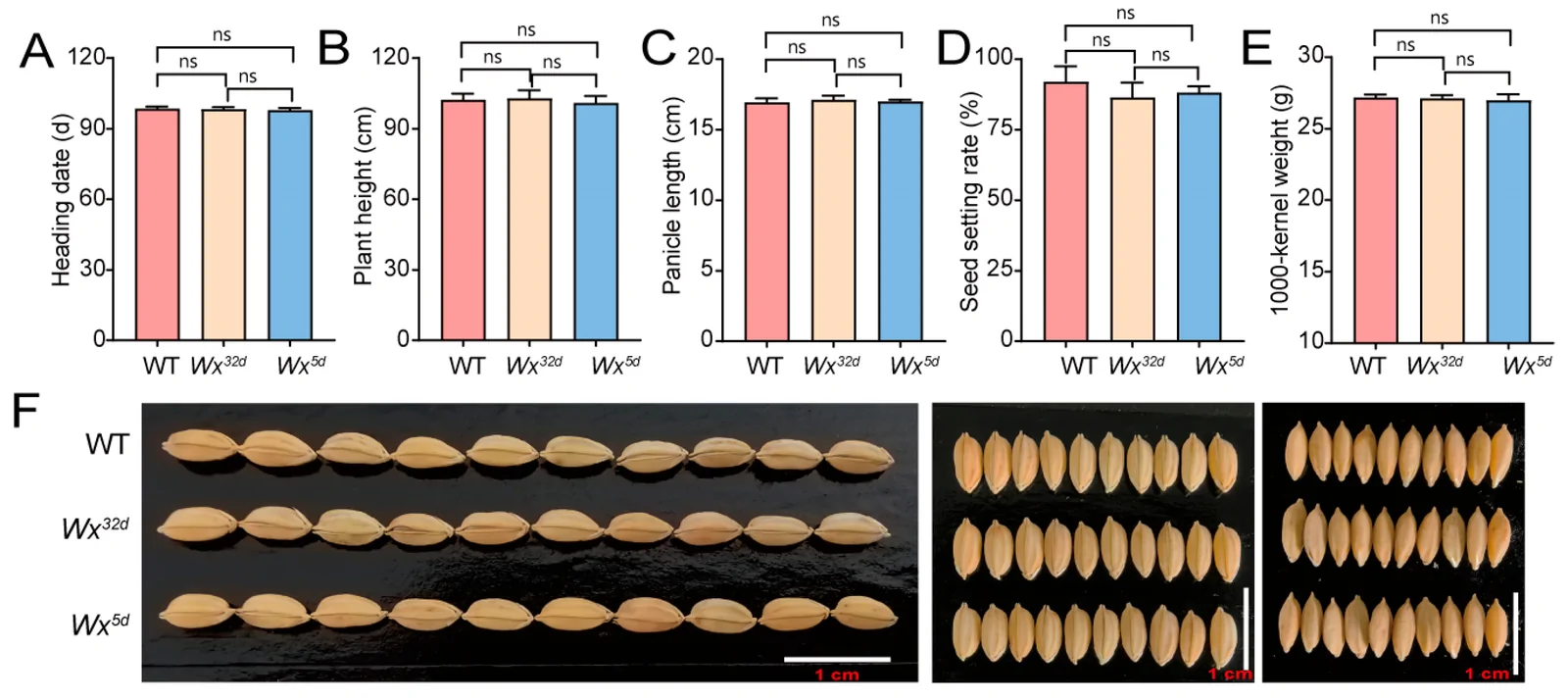
Comparison of agronomic traits between edited lines (*Wx^32d^*, *Wx^5d^*) and wild-type (*Wx^b^*). (**A**–**E**) Statistical comparison of heading date, plant height, panicle length, seed setting rate, and 1000-grain weight between mutants and WT. (**F**) Grain length, grain width, and grain thickness. Values represent mean ± SD (*n* = 30 plants per line, 3 replicates). ns: not significant (one-way ANOVA, *p* > 0.05). The scale bar in (**F**) represents 1 cm.

Analysis of variance (ANOVA) of eight core agronomic traits, including plant height, growth period, seed setting rate, panicle length, 1000-grain weight, grain length, grain width, and grain thickness, revealed no statistically significant differences (*p* > 0.05 for all traits, one-way ANOVA; Table 3) between the edited lines and the wild-type, although a mild reduction in seed setting rate was observed in *Wx^32d^* (86.43% versus 91.96%, *p* = 0.14) (Figure 3). This mild, non-significant reduction is reported as a trend that warrants close monitoring in future multi-environment trials. These phenotypic results suggest that PE-mediated targeted editing improves grain quality parameters without exerting significant adverse impacts on the agronomic traits evaluated in this study.

## 3. Discussion

With increasing consumer demand for high-quality rice, grain quality improvement remains a primary priority in rice breeding. Natural allelic variation at the *Wx* locus has been extensively utilized for rice quality improvement [7,8,9]. Nevertheless, available variants mainly originating from the coding region are approaching exhaustion; furthermore, these alleles usually induce excessive reduction in AC and possess limited potential for precise phenotypic modulation [18]. In this context, targeted editing of putative promoter segments represents a promising alternative strategy to expand functional allelic diversity for quality improvement.

Our strategy builds directly on the pioneering demonstration by Huang et al. [18] that CRISPR/Cas9-mediated promoter editing of *Wx* can fine-tune AC in rice. The present work extends that precedent in three respects. First, prime editing installs predefined deletions without double-strand breaks or donor DNA, whereas CRISPR/Cas9 relies on stochastic NHEJ products that must be recovered through large-scale screening. Second, the targeted PRR interval immediately upstream of the TSS yielded alleles that are absent from both the 3032 natural accessions surveyed here and the allele series reported by Huang et al. [18] and in subsequent *Wx* promoter-editing studies [27,28]. Third, the 5 bp and 32 bp deletions recovered from a single pegRNA experiment constitute an internal allelic gradient (AC reductions of 2.77 and 3.95 percentage points), enabling direct structure-function comparison within the same genetic background.

In this study, PE was deployed to modify the bioinformatically predicted PRR within the *Wx* promoter. Two deletion alleles were recovered: the designed *Wx^5d^* carrying a precise 5 bp deletion, and the unanticipated *Wx^32d^* carrying a 32 bp deletion. The two alleles reduced grain AC by 2.77 and 3.95 percentage points, respectively, with the magnitude of AC reduction correlating positively with deletion size. The modified lines exhibited improved taste values, longer gel consistency, and RVA profiles with higher breakdown viscosity and more negative setback, while major agronomic traits remained largely stable under field conditions.

The 32 bp deletion represents an unplanned yet informative allelic variant, presumably generated via DSB-mediated non-homologous end joining (NHEJ) upon nickase activity. Arising spontaneously during independent T_0_ regeneration instead of being pre-designed, this serendipitous allele facilitates the evaluation of phenotypes driven by extensive promoter modifications and complements the engineered 5 bp deletion. The nick-sgRNA in the PE3b system may occasionally trigger target-site DSBs; apart from desired precise small edits, subsequent NHEJ repair could lead to large fragment deletions. Since these structural rearrangements are restricted to the target editing interval, such events are defined as on-target unintended edits rather than off-target mutations. Together, the identification of these distinct alleles suggests prime editing can generate a broader spectrum of variants beyond preplanned modifications, which may expand its applications in rice germplasm innovation.

The transformation efficiency was 7.50% (15 independent T_0_ plants from 200 calli; Table 1). The mutation efficiency observed here (66.67%) is higher than several previous PE reports in rice [29] and likely reflects the combined effects of the PE3b configuration, in which the additional nicking sgRNA biases repair toward the edited strand, and target-intrinsic features: the spacer lies within a GC-rich, transcriptionally active core-promoter region near the TSS where open chromatin may favor Cas9 accessibility, and the installed edit is a short, deletion-only change, a class that prime editors write with relatively high efficiency. Because these data derive from a single locus in a single cultivar, this efficiency should not be extrapolated to other targets; multi-locus comparisons will be required to disentangle the respective contributions of vector architecture and target-site features.

The edited target interval spans −45 to −7 bp upstream of the *Wx* TSS, coinciding with the PRR-like interval delineated by our sequence analysis. Sequence deletions within this region were accompanied by moderately reduced *Wx* transcript levels and lower OsGBSSI protein accumulation, which paralleled the observed declines in AC. For descriptive convenience, the target region was divided into two subdomains, PRR1 (−45 to −19) and PRR2 (−11 to −7). Notably, current data are insufficient to establish a definite causal relationship between specific sequence alterations and transcriptional regulation, and the functional independence of PRR1 and PRR2 remains unconfirmed. Future studies employing promoter-reporter assays and electrophoretic mobility shift assays (EMSAs) will be required to dissect the individual contributions of these sequence segments to *Wx* transcription or translation. Chromatin immunoprecipitation followed by qPCR (ChIP-qPCR) will additionally be employed to identify transcription factors occupying these sequences.

Neither of the two edited alleles was detected in a panel of 3032 rice accessions, further supporting that promoter editing can generate novel genetic variants that are rarely present in natural germplasm. In particular, the *Wx^32d^* allele achieves an AC level of 12.40%, which is comparable to that of conventional soft rice, while retaining the superior agronomic performance of the elite *japonica* cultivar Huaidao 5, although the mild, statistically non-significant reduction in seed setting rate observed in *Wx^32d^* warrants close monitoring in multi-environment trials before the line is advanced in breeding programs. These features make the two novel alleles promising intermediate materials for future rice quality breeding.

The targeted PRR is highly conserved among the 3032 surveyed accessions, which span both japonica and indica subgroups, indicating that the same pegRNA can in principle be deployed in other elite backgrounds. Editing efficiency and the magnitude of AC reduction may nevertheless vary with genetic background, depending on the recipient cultivar’s basal *Wx* expression level and its pre-existing *Wx* allele. Transfer of *Wx^32d^* and *Wx^5d^* into additional japonica and indica cultivars, by both crossing and re-editing, is underway to test the generalizability of this strategy. In these crosses, *Wx^32d^* and *Wx^5d^* are being used as donor parents for the low-AC alleles, and the performance of the resulting backcross/derivative progeny will directly test the breeding value of the two alleles and the stability of their effects across genetic contexts.

It should be recognized that *Wx* is a highly pleiotropic gene whose documented effects extend beyond amylose biosynthesis to gel consistency, gelatinization temperature, RVA pasting properties, grain transparency and chalkiness [27], 1000-grain weight, spikelet fertility, seed lysophosphatidylcholine (LPC) content [30], and milled-rice thiamin accumulation [31]. The present evaluation covered a subset of this network (AC, instrumental taste value, gel consistency, RVA pasting properties, grain appearance, and eight agronomic traits) in a single environment. Gelatinization temperature, a trained sensory panel, plot-level yield assessment, and targeted grain metabolomics are therefore mandatory endpoints of the ongoing multi-environment trials before the edited lines can be confirmed as breeding-ready germplasm.

Several limitations of this study should be noted. All field evaluations were conducted at a single site (Yangzhou, Jiangsu) during a single growing season (May–October 2025). Multi-location and multi-year trials are necessary to assess the environmental stability of the observed phenotypes. Grain eating quality was evaluated by instrumental taste value, gel consistency, and RVA pasting profiles, without complementary sensory evaluation. In addition, off-target assessment was limited to several high-risk loci rather than genome-wide screening. Similarly, grain metabolomics (including LPC and thiamin) and standardized human sensory panels evaluating texture, stickiness, glossiness, and hardness of cooked rice have not yet been performed. Multi-location field trials, systematic sensory assessments, untargeted grain metabolomics, and comprehensive genome-wide off-target analysis (whole-genome sequencing) will be necessary to fully validate the stability and reliability of the edited phenotypes in future studies.

In summary, PE-mediated editing of the *Wx* promoter PRR can generate novel low-AC alleles with gradient regulatory effects and minimal agronomic penalties among the traits evaluated. This promoter-editing strategy shows potential for creating fine-tuned quality germplasm and may be adaptable for precision modification of other quality-associated genes in rice.

## 4. Materials and Methods

### 4.1. Plant Materials

The elite *japonica* rice cultivar Huaidao 5 was used as the transformation material in this study. Huaidao 5 exhibits excellent agronomic performance and carries the conventional *Wx^b^* allele with a moderate AC of approximately 15–16%, which is suitable for subsequent grain quality modification.

### 4.2. Prime Editing Workflow

#### 4.2.1. Target Design and Off-Target Evaluation

Our sequence analysis of the *Wx* promoter delineated a PRR-like interval located −45 to −7 bp upstream of the TSS, which falls within the typical distribution window of PRRs (Y patches) reported for plant core promoters (−50 to +50 bp relative to the TSS) [19,32]. A continuous 5 bp cytosine stretch (CCCCC, chr6:1765377-1765381) located at −11 to −7 bp was selected as the initial editing target to explore whether sequence alterations in this region could cause phenotypic changes, whereas the 32 bp deletion (*Wx^32d^*) was recovered as an unintended editing outcome from independent transformation events. Partial deletions were designed to achieve fine-tuned reduction in AC rather than gene abolition.

Two distinct homozygous genotypes were identified from T_0_ to T_2_ generations: one carrying a 32 bp deletion removing both the upstream segment (−45 to −19) and the targeted sequence (−11 to −7), and the other harboring only the precise 5 bp deletion at −11 to −7. For descriptive purposes, the upstream deleted segment (−45 to −19) and the 5 bp segment (−11 to −7) were retrospectively designated PRR1 and PRR2, respectively.

The NGG protospacer adjacent motif (PAM) suitable for Cas9 nickase recognition was determined according to the target sequence characteristics. Potential off-target effects were evaluated using Cas-OFFinder (v2.4; http://www.rgenome.net/cas-offinder/ (accessed on 5 February 2024)) with parameters: seed length = 12 nt, maximum DNA bulge size = 2, maximum RNA bulge size = 2, and maximum mismatches = 3. Potential off-target loci were identified across the rice genome (Table A1). The top 5 predicted off-target sites were validated by Sanger sequencing in homozygous T_2_ edited lines (Table A2).

The PE3b system was employed in this study. The prime editing guide RNAs (pegRNAs) were designed to contain a spacer sequence fully complementary to the target DNA, a primer binding site (PBS) that binds the 3′ end of the target DNA to initiate reverse transcription, and a reverse transcription template (RTT) harboring the designed 5 bp deletion sequence. An additional nicking sgRNA was also designed to nick the non-edited strand for improved editing efficiency. Detailed sequence information is listed in Table 4.

#### 4.2.2. Construction of Gene Editing Vectors

Synthesis of Target Gene Amplification Primers

Primers for PCR amplification were designed as follows:

QC5bp-L:

5′-cagtGGTCTCatgcatgaaggaatggtgagagagggtttcag-3′

QC5bp-R:

5′-cagtGGTCTCatcaagcaccgactcggtgccac-3′

PCR amplification, sequencing, and enzymatic digestion-ligation

PCR amplification was performed in a 50 μL PCR reaction system containing 2 μL of forward primer (10 μM), 2 μL of reverse primer (10 μM), 25 μL of 2× Taq PCR Mix, 20 μL of ddH_2_O, and 1 μL of template DNA. Cycling conditions: initial denaturation at 94 °C for 5 min, followed by 30 cycles of denaturation at 94 °C for 30 s, annealing at 50 °C for 45 s, and extension at 72 °C for 22 s, with a final extension at 72 °C for 10 min and a final hold at 16 °C for 30 min.

Amplified products were gel-purified and verified by sequencing, followed by one-pot digestion-ligation with the pHU-PE-Lncas9(K34) vector using the BsaI/Eco31I restriction enzyme. The pHU-PE-Lncas9(K34) vector is a PE3b-compatible backbone vector that expresses the nCas9(H840A)-M-MLV reverse transcriptase fusion protein. The digestion-ligation procedure was conducted as below: incubation at 37 °C for 20 min, five cycles alternating between 37 °C for 10 min and 20 °C for 10 min, terminal digestion at 37 °C for 20 min, and enzyme inactivation at 80 °C for 5 min. Ligated plasmids were transformed into competent *E. coli* cells through heat shock at 42 °C for 90 s. Transformed cells were recovered in liquid LB medium at 37 °C for 1 h and cultured on kanamycin-containing LB agar plates. Positive recombinant clones were screened via colony PCR with primer set PE-max-F (5′-atcttcaaaagtcccacatcg-3′) and PE-max-R (5′-cattcgccatgccgaagcat-3′), and accurate insertion was further confirmed by Sanger sequencing.

#### 4.2.3. Genetic Transformation

Plasmids were electroporated into *Agrobacterium tumefaciens* strain EHA105, and transformants were verified by PCR.

Rice seeds without mold spots and with normal germ pores were selected and surface-sterilized sequentially with 75% ethanol for 1 min and 15% sodium hypochlorite for 20 min, followed by three rinses with sterile water. Sterilized seeds were inoculated on callus induction medium. Calli were induced at 26 °C (16 h light/8 h dark) for 20 days, and embryogenic calli were subcultured every 10–12 days for 25–30 days.

For *Agrobacterium*-mediated transformation, embryogenic calli were pre-cultured for 3 days and infected with an *Agrobacterium* suspension adjusted to OD_600_ = 0.2 for 10–15 min, followed by co-cultivation at 22 °C in the dark for 3 days on co-cultivation medium supplemented with 100 μM acetosyringone. Calli were selected on hygromycin B (50 mg/L) and carbenicillin (300 mg/L) for two rounds (2 weeks each), differentiated at 26 °C (16 h light/8 h dark) for 25–30 days, and rooted for 10–15 days before transplantation to the field (Table A4). Genomic DNA was extracted from leaf tissues, and PCR analysis was performed to identify positive transgenic plants and determine their editing types.

### 4.3. Analysis of Wx Gene Expression and AC in Transgenic Lines

T_0_ positive seedlings were cultivated hydroponically and then transplanted to the field under conventional field management. Mature seeds were harvested and planted in Hainan to propagate the T_1_ generation, with 30 individual plants retained for each transgenic line. Homozygous edited plants were identified via direct sequencing of target amplicons. Developing grains at 15 days after flowering (DAF) were sampled for quantitative real-time PCR (qRT-PCR) to detect the transcriptional level of the *Wx* gene. After full ripening, grain AC and GBSSI protein accumulation were determined. An automatic rice taste analyzer (STA1B, Satake Corporation, Hiroshima, Japan) was adopted to test taste value for evaluating cooked rice palatability.

#### 4.3.1. AC Measurement

Milled rice grains were ground and passed through a 100-mesh sieve. Exactly 0.1000 g of rice flour was subjected to alkaline dissolution and iodine colorimetric assay following the simplified amylose quantification procedure described by Juliano [33]. A standard curve was established using a rice starch matrix blended with gradient potato amylose standards (Sigma-Aldrich, St. Louis, MO, USA, A0512) at proportions of 0%, 5%, 10%, 15% and 20% (*w*/*w*). The standard curve showed excellent linear fitting (*R^2^* > 0.999, *y* = 0.045*x* + 0.012), in which *y* represented absorbance at 620 nm, and *x* stood for amylose content (%).

#### 4.3.2. qRT-PCR Detection of *Wx* Transcription

Total RNA was extracted from 15 DAF grains using a commercial plant RNA extraction kit (Vazyme, Nanjing, China, RM201), followed by reverse transcription to synthesize first-strand cDNA. The housekeeping gene *OsUBQ5* was used for expression normalization, with primer sequences: forward 5′-GACTACAACATCCAGAAGGAGTC-3′, reverse 5′-TCATCTAATAACCATTCGATTTTC-3′ [34]. Specific primers for the *Wx* gene were designed via Primer3.0 (https://primer3.ut.ee/ (accessed on 14 March 2025)): forward 5′-AATCAGTGAGATCACGCCCAG-3′, and reverse 5′-TTGGCAATAAGCCACACACA-3′. Melting curve profiling and agarose gel electrophoresis were used to verify the amplification specificity of target fragments. The relative gene expression level was calculated with the 2^−ΔΔCt^ algorithm.

#### 4.3.3. Western Blot Quantification of GBSSI Protein

Total storage protein was isolated from mature brown rice using P-PER Plant Protein Extraction Reagent (Thermo Scientific, Waltham, MA, USA) in accordance with the manufacturer’s instructions. GBSSI protein abundance was characterized by Western blot. The primary antibody was rabbit anti-GBSSI polyclonal antibody (Agrisera, Vannas, Sweden, AS09 525, dilution ratio 1:1000), and the secondary antibody was HRP-labeled goat anti-rabbit IgG (Thermo Scientific, Waltham, MA, USA, 1:5000). Tubulin (Sigma-Aldrich, St. Louis, MO, USA, T5168, T5168, 1:5000) was applied as the internal reference for protein loading.

Band intensities were quantified in ImageJ v1.53. Rolling-ball background subtraction (radius = 50 pixels) was applied to raw 16-bit TIFF images prior to gray-value measurement. Serial dilution assays verified that all band signals fell within the linear detection range. GBSSI signal intensities were normalized to the tubulin loading control, and relative protein abundance was calculated as fold change compared with the wild-type.

#### 4.3.4. Gel Consistency Measurement

Gel consistency was determined. Briefly, 100 mg of milled rice flour was mixed with 0.2 mL of 0.2% thymol blue and 2.0 mL of 0.2 M KOH, vortexed, incubated at room temperature for 8 min, and then cooled in ice-water for 20 min, followed by standing at 25 °C for 2 h. The length of the migrated gel was measured in millimeters.

#### 4.3.5. RVA Pasting Properties

Pasting properties were measured with a Rapid Visco Analyser (Newport Scientific, Warriewood, Australia) using the standard 12 min temperature profile. Milled rice flour (3.00 g, 12% moisture basis) was dispersed in 25.0 g of distilled water. Peak viscosity (PV), hot paste viscosity (HV), breakdown (BV = PV − HV), final viscosity (FV), set-back (SV = FV − HV), setback (Setback = FV − PV), peak time (PT) and pasting temperature (PTemp) were recorded according to the manufacturer’s software.

### 4.4. Investigation of Major Agronomic Traits in Transgenic Lines

Agronomic traits were investigated and compared between homozygous edited lines and the control, including heading date, plant height, panicle length, seed setting rate, 1000-grain weight, grain length, grain width, and grain thickness. The field trial was conducted at Yangzhou, Jiangsu (32.4° N, 119.4° E), May–October 2025. Transplanted June 15, harvested October 20. Each line contained 30 plants, arranged in a randomized block design with three field replicates. The average seasonal temperature was 26.5 °C and cumulative precipitation reached 850 mm throughout cultivation.

### 4.5. Statistical Analysis

All phenotypic, physiological, molecular and agronomic trait measurements in this study were conducted with three biological replicates, and each biological replicate contained three technical replicates.

All quantitative data were analyzed by one-way analysis of variance (ANOVA). Tukey’s HSD post hoc test was used for pairwise comparisons among wild-type (*Wx^b^*), *Wx^32d^* and *Wx^5d^* genotypes. Data are presented as mean ± SD. Differences with *p* < 0.05 were considered statistically significant, and *p* < 0.01 indicated highly significant differences. All statistical analyses were conducted using SPSS 26.0 (IBM, USA).

## 5. Conclusions

This study generated two novel low-amylose *Wx* alleles, *Wx^32d^* and *Wx^5d^*, through PE targeting the promoter region of the rice *Wx* gene. Field planting trials showed that these edited lines exhibited lower AC, longer gel consistency, and RVA profiles consistent with softer paste, with their grain appearance intact and major agronomic traits basically consistent with wild-type plants. The −45 to −7 region of the *Wx* promoter has great potential as a key editing locus for precise rice quality breeding, while its specific regulatory role in starch synthesis and grain quality formation still needs further in-depth investigation. The overall results confirm that PE is an effective approach to develop improved rice germplasm. This technical method can precisely optimize rice grain quality without causing obvious negative effects on the agronomic traits evaluated. Among the two mutated alleles, *Wx^32d^* can effectively reduce grain AC and optimize rice taste performance, making it a desirable intermediate germplasm resource for high-quality breeding of *japonica* rice based on the Huaidao 5 genetic background. Given the influence of environmental factors on crop traits, extended field tests across multiple locations and consecutive years are essential to confirm the stability of the improved quality traits of these edited lines. Moreover, further research is also required to verify whether this promoter editing strategy can be universally applied to quality improvement in other excellent rice varieties. Ongoing crossing and re-editing experiments are being conducted to transfer *Wx^32d^* and *Wx^5d^* into additional japonica and indica backgrounds, thereby evaluating the breeding value, inheritance stability, and phenotypic effects of these alleles across diverse genetic contexts.

## Figures and Tables

**Table 1:** Statistics of prime editing transformation and mutation types in T_0_ generation.

Item	Number	Ratio
Transformed calli	200	–
Regenerated independent T_0_ transgenic plants	15	Transformation efficiency: 7.50%
T_0_ plants with *Wx* promoter mutations	10	Mutation efficiency: 66.67%
32 bp deletion mutant lines	4	40.00% of mutant plants
Precise 5 bp deletion mutant lines	3	30.00% of mutant plants
Other indel mutants (discarded)	3	30.00% of mutant plants

**Table 2:** Gel consistency and key RVA parameters of wild-type and edited rice lines.

Parameter	WT	*Wx^32d^*	*Wx^5d^*
PV (mPa·s)	3528.50 ± 21.79 a	3262.33 ± 27.42 b	2949.75 ± 37.83 c
HV (mPa·s)	2926.67 ± 35.09 a	2255.00 ± 35.77 b	2319.50 ± 53.03 b
BV (mPa·s)	601.83 ± 43.20 b	1007.33 ± 19.22 a	630.25 ± 15.20 b
FV (mPa·s)	3768.67 ± 2.02 a	2794.33 ± 35.57 b	2849.00 ± 55.15 b
SV (mPa·s)	842.00 ± 34.83 a	539.33 ± 7.69 b	529.50 ± 2.12 b
Setback (mPa·s)	+240.17 ± 19.85 a	−468.00 ± 13.44 c	−100.75 ± 17.32 b
PT (min)	6.24 ± 0.04 a	5.51 ± 0.04 c	5.83 ± 0.05 b
PTemp (°C)	71.78 ± 0.04 c	73.38 ± 0.01 b	74.29 ± 0.02 a

Values are means ± SD (*n* = 3 biological replicates). Different lowercase letters within a column indicate significant differences among lines (one-way ANOVA, Tukey’s HSD, *p* < 0.05). PV, peak viscosity; HV, hot-paste viscosity (trough); BV, breakdown viscosity (PV − HV); FV, final viscosity; SV, setback viscosity (FV − HV); setback, setback (FV − PV); PT, peak time; PTemp, pasting temperature. Viscosity values are in mPa·s; PT in min; PTemp in °C. The corresponding RVA profiles are shown in Figure 2H.

**Table 3:** Agronomic trait performance of wild-type and edited rice lines.

Traits	*Wx^b^* (*WT*)	*Wx^32d^*	*Wx^5d^*	Significant Different
Heading date (days)	98.50 ± 1.00	98.25 ± 0.96	98.00 ± 0.82	ns
Plant height (cm)	102.40 ± 2.50	102.92 ± 3.53	101.00 ± 3.00	ns
Panicle length (cm)	16.93 ± 0.28	17.13 ± 0.28	17.00 ± 0.14	ns
Seed setting rate (%)	91.96 ± 5.57	86.43 ± 5.30	88.15 ± 2.22	ns
1000-grain weight (g)	27.18 ± 0.22	27.14 ± 0.21	26.98 ± 0.45	ns
Grain length (mm)	7.38 ± 0.02	7.39 ± 0.03	7.40 ± 0.04	ns
Grain width (mm)	3.09 ± 0.05	3.10 ± 0.07	3.07 ± 0.14	ns
Grain thickness (mm)	2.33 ± 0.09	2.30 ± 0.05	2.30 ± 0.02	ns

Values are mean ± SD (*n* = 30). No significant differences were detected among lines for any trait (one-way ANOVA, *p* > 0.05).

**Table 4:** Sequence information for pegRNA and nicking sgRNA components.

Component	Sequence (5′→3′)	Length	Function
Spacer	TGAAGGAATGGTGAGAGAGG	20 nt	Target DNA recognition
PAM	GGG	3 nt	Cas9 nickase recognition
3′ extension (PBS)	ACACCACCAT	10 nt	Primer binding site for reverse transcription
3′ extension (RTT)	CTCTCACCATTC	12 nt	Reverse transcription template
Nicking gRNA	TGTCATCGTCGACACCGGCCGGG	23 nt	Nicking non-edited strand to enhance editing efficiency

## Data Availability

The original contributions presented in this study are included in the article/Appendix A. Further inquiries can be directed to the corresponding authors.

## Abbreviations

AC	amylose content
ANOVA	analysis of variance
BV	breakdown viscosity
DAF	days after grain filling
DSBs	double-strand breaks
EMSA	electrophoretic mobility shift assay
GC	gel consistency
NHEJ	non-homologous end joining
OsGBSSI	granule-bound starch synthase I
PBS	primer binding site
PE	prime editing
pegRNAs	prime editing guide RNAs
PRR	pyrimidine-rich region
qRT-PCR	quantitative real-time PCR
RTT	reverse transcription template
RVA	Rapid Visco Analyser
TSS	transcription start site

## Appendix A

**Table A1:** Off-target prediction and validation for the pegRNA used in this study.

Site	Chr.	Position (IRGSP-1.0)	Strand	Off-Target Sequence (5′→3′) (Lowercase = Mismatch)	PAM	Mismatches	DNA Bulge	Gene Context	Mutation Detected?
target	Chr06	1,765,500	+	TGAAGGAATGGTGAGAGAGG	GGG	0	0	*Wx* promoter	Yes
OT1	Chr08	3,334,573	+	TGAAGGCAATGGaGAGgGAGG	AGG	2	1	Intergenic	No mutation detected
OT2	Chr08	10,507,762	−	aGAAGGAAgGGAGGAGAGAGG	AGG	3	1	Intergenic	No mutation detected
OT3	Chr08	14,036,587	+	TGAAaGAAgGGaGAGAGAGG	GGG	3	1	Intron	No mutation detected
OT4	Chr08	15,062,669	+	gGgAGGAGAaGGTGAGAGAGG	AGG	3	1	Intron	No mutation detected
OT5	Chr08	26,617,244	−	aGAAGGCAATGGTGAGcGAGa	TGG	3	1	Intron	No mutation detected
OT6	Chr08	27,805,714	−	gGAAGGGAATGGcGAGgGAGG	CGG	3	1	Intergenic	——
OT7	Chr08	28,194,141	−	aGAAGGAAgGGaGGAGAGAGG	AGG	3	1	Intergenic	——

**Table A2:** The primers for off-target validation.

Site	Forward	Reverse
OT1	ACTGTTGGGAGGTCGAAATG	AGTAATTCGGTCAGTTCGGTTAT
OT2	GGGTCTAATTTGGTGAGGGTT	ACTGGGAAAGGATGCTTCTG
OT3	TTTGTACGGTCCAAGGATGTC	CTGAGAAGCTTTCGCGTATCT
OT4	TGCAGCTTCCTCTTAACCATAA	TCACCAACTTGAGAAAGGAAGT
OT5	GCACTAGAGCTCGATGTTTCA	ACCTCTCCCAACGGATAGT

**Table A3:** Quantitative measurements of grain chalkiness and translucency in wild-type and edited lines.

Traits	WT	*Wx* *^32^* *^d^*	*Wx* *^5^* *^d^*	*p*-Value
Chalkiness	0.2316	0.1969	0.2277	0.7363 ns
	0	0.3368	0	
	0	0	0.2915	
	0	0.3559	0.2813	
	0.2112	0	0	
	0.2071	0	0	
	0.4229	0	0.1884	
	0	0.1788	0.1141	
	0.3753	0	0	
	0.1280	0	0.1718	
	0.3460	0	0.2392	
	0.1175	0.1954	0	
	0.0618	0	0.4845	
	0.3385	0	0	
	0	0.2048	0.1941	
	0	0	0	
	0.1808	0.4277	0.1962	
	0	0	0.1667	
	0.1560	0.2333	0	
	0	0.2586	0.2552	
	0	0	0	
	0.2308	0	0	
	0	0	0	
	0.1994	0	0	
	0	0.1429	0.2968	
	0	0.3117	0	
	0	0	0.3084	
	0.2204	0	0	
	0	0.0712	0	
	0.3151	0	0	
Translucency	2	2	2	ns

ns, not significant (*p* > 0.05).

**Table A4:** The compositions of the media.

Medium	Component	Concentration
Callus induction medium	MS basal salts	4.43 g/L
	Sucrose	30 g/L
	2,4-D	2.0 mg/L
	Phytagel	3.0 g/L
	pH	5.8
Selection medium	MS basal salts	4.43 g/L
	Sucrose	30 g/L
	2,4-D	2.0 mg/L
	Hygromycin B	50 mg/L
	Carbenicillin	300 mg/L
	Phytagel	3.0 g/L
	pH	5.8
Differentiation medium	MS salts	4.43 g/L
	Peptone	500 mg/L
	Proline	500 mg/L
	Glutamine	300 mg/L
	Maltose	30 g/L
	Thidiazuron (TDZ)	0.2 mg/L
	or Kinetin	2.0 mg/L
	Phytagel	3.0 g/L
	pH	5.8
Rooting medium	1/2 MS salts	2.215 g/L
	Indole-3-butyric acid (IBA)	0.5 mg/L
	Sucrose	15 g/L
	Phytagel	3.0 g/L
	pH	5.8
